# Detection of diagnostic somatic copy number alterations from cerebrospinal fluid cell-free DNA in brain tumor patients

**DOI:** 10.1186/s40478-024-01887-9

**Published:** 2024-11-20

**Authors:** Svenja Klinsing, Julia Beck, Katharina J. Weber, Kirsten Bornemann-Kolatzki, Mareike Dettki, Hans Urban, Bastian Roller, Kai U. Chow, Henning Reis, Peter J. Wild, Ekkehard Schuetz, Philipp Euskirchen, Joachim P. Steinbach, Michael W. Ronellenfitsch, Patrick N. Harter, Pia S. Zeiner

**Affiliations:** 1 Dr. Senckenberg Institute of Neurooncology, University Hospital, Goethe University Frankfurt, Frankfurt, Germany; 2 Department of Neurology, University Hospital, Goethe University Frankfurt, Frankfurt, Germany; 3 Chronix Biomedical, Oncocyte, Göttingen, Germany; 4 Institute of Neurology (Edinger-Institute), University Hospital, Goethe University Frankfurt, Frankfurt, Germany; 5 Frankfurt Cancer Institute (FCI), Goethe University Frankfurt, Frankfurt, Germany; 6 University Cancer Center (UCT), University Hospital, Goethe University Frankfurt, Frankfurt, Germany; 7 German Cancer Research Center (DKFZ) Heidelberg, Germany and German Cancer Consortium (DKTK), Partner Site Frankfurt/Mainz, Frankfurt, Germany; 8 Ambulantes Krebszentrum, Frankfurt, Germany; 9 Dr. Senckenberg Institute of Pathology, Goethe University Frankfurt, University Hospital, Frankfurt, Germany; 10 Department of Neuropathology, Charité-Universitätsmedizin Berlin, Corporate Member of Freie Universität Berlin Und Humboldt Universität Zu Berlin, Berlin, Germany; 11 German Cancer Consortium (DKTK), Partner Site Berlin, A Partnership Between DKFZ and Charité - Universitätsmedizin Berlin, Berlin, Germany; 12 Center for Neuropathology and Prion Research, Faculty of Medicine, LMU Munich, Munich, Germany; 13 German Cancer Consortium (DKTK), Partner Site MunichA Partnership Between DKFZ and University, University Hospital, LMU Munich, Munich, Germany

**Keywords:** Cell-free DNA, Cerebrospinal fluid, Liquid biopsy, Next-generation sequencing, Brain tumor

## Abstract

The gold standard for precise diagnostic classification of brain tumors requires tissue sampling, which carries relevant procedural risks. Brain biopsies often have limited sensitivity and fail to address tumor heterogeneity, because small tissue parts are being examined. This study aims to explore the detection and quantification of diagnostically relevant somatic copy number aberrations (SCNAs) in cell-free DNA (cfDNA) extracted from cerebrospinal fluid (CSF) in a real-world cohort of patients with defined brain tumor subtypes. A total of 33 CSF samples were collected from 30 patients for cfDNA extraction. Shallow whole-genome sequencing was conducted on CSF samples containing > 3ng of cfDNA and corresponding tissue DNA from nine patients. The sequencing cohort encompassed 26 samples of 23 patients, comprising 12 with confirmed CNS cancer as compared to 11 patients with either ambiguous CNS lesions (n = 5) or non-cancer CNS lesions (n = 6). After mapping and quality filtering SCNAs were called by depth-of-coverage analyses with a binning of 5.5 Mbp. SCNAs were exclusively identified in CSF cfDNA from brain tumor patients (10/12, 83%). In tumor patients, SCNAs were detectable in cfDNA from all patients with, but also in five of seven patients without tumor cells detected by CSF cytopathology. A substantial number of shared SCNAs were traceable between tissue and CSF in matched pair analyses. Additionally, some SCNAs unique to either CSF or tissue indicating spatial heterogeneity or tumor evolution. Also, diagnostically relevant genomic alterations as well as essential and desirable SCNAs as implemented in the current WHO classification of CNS tumors for certain primary brain tumor subtypes were traceable. In summary, this minimally invasive cfDNA-based LB approach employing shallow whole genome sequencing demonstrates potential for providing a molecularly informed diagnosis of CNS cancers, mapping tumor heterogeneity, tracking tumor evolution, and surveilling tumor patients. Further prospective trials are warranted.

**Supplementary Information:**

The online version contains supplementary material available at 10.1186/s40478-024-01887-9.

## Introduction

Accurate diagnostic evaluation of lesions in the central nervous system (CNS) usually necessitates invasive procedures like stereotactic brain biopsy. However, CNS tissue sampling poses substantial risks, particularly in eloquent CNS areas or in patients with compromised performance states. To address these challenges, less invasive approaches provide an opportunity to mitigate complications and allow for repeated sampling, which is particularly advantageous in clinical scenarios when nonsurgical treatment is the preferred option or for longitudinal disease monitoring.

The term liquid biopsy (LB) refers to diagnostic tools that allow for the minimal invasive assessment of parameters from body fluids and various cellular and cell-free (cf) analytes can be investigated simultaneously in a single sample. The RANO (Response Assessment in Neuro-Oncology) group has recently taken the initiative to review the utilization of LBs in both primary [1] and secondary [2] brain tumors as well as CNS lymphomas [3] with the goal to assess the feasibility of LB approaches and their potential integration into clinical trials or neuro-oncological practice. LBs derived from cerebrospinal fluid (CSF) offer advantages over blood LBs in patients with brain tumors, as they exhibit higher sensitivity in detecting brain tumor-derived analytes such as cfDNA [1, 2, 4–8]. Currently, routine CSF diagnostics primarily serves the purpose of clarifying non-neoplastic differential diagnoses or detecting tumor cells in case of leptomeningeal disease (LMD) due to its prognostic relevance and urgent therapeutic implications. This includes the analysis of indirect LMD parameters (increased opening pressure, barrier dysfunction, or lactate elevation), direct LMD parameters (detection of tumor cells through cytology), or non-cellular tumor markers. However, in contrast to the advances in tissue diagnostics, molecular pathological CSF profiling is still limited to individual applications such as clonality assays or the detection of hotspot mutations to support specific diagnoses, such as diffuse large B-cell lymphoma of the CNS (CNS-DLBCL) [9]. Given the importance of accurate and timely molecular pathological profiling, now considered a state-of-the-art for tissue diagnostics in neuro-oncology [10, 11] and facilitated by emerging technologies allowing for same-day or even intraoperative molecularly informed diagnosis [12–15], there exists vast potential to explore the applications of LBs in neuro-oncology [1, 4]. Tumor-derived cfDNA emerges as a particularly promising analyte, with various features available for evaluation [16–18]. Aneuploidy, represented by somatic copy number aberrations (SCNAs), is a fundamental characteristic of tumor-derived DNA detectable in cfDNA for example through next-generation sequencing (NGS). Further, quantifying cfDNA aneuploidy via chromosomal number instability (CNI) scoring proved to be promising for predicting early responses to immunotherapy in advanced non-CNS tumors. The approach surpasses merely measuring the total tumor cfDNA concentration as the CNI score serves as a metric for tumor-derived copy number instability and does not necessitate prior knowledge of somatic tumor mutations [19]. Taken together, SCNA profiling has gained immense significance for diagnostic classification of brain tumors. Among the approximately 100 different subtypes of brain tumors according to the current WHO classification, particular SCNAs are incorporated as essential criteria in 12 subtypes and as desirable criteria in 13 subtypes [11]. For instance, detecting the 1p/19q co-deletion aids in diagnosing oligodendrogliomas, while + 7/ − 10 copy number changes in IDH-wildtype diffuse astrocytomas enable the diagnosis of glioblastoma. The CDKN2A/B homozygous deletion is an essential diagnostic alteration in diagnosing CNS WHO grade 4 in IDH-mutant astrocytomas and desirable for pleomorphic xanthoastrocytoma [11]. Moreover, certain SCNAs were identified as indicators for predicting the prognostic outcome [11, 20, 21]. Besides brain tumors, SCNAs also play a role in the context of extracranial tumors that exhibit a strong propensity for CNS or CSF involvement. The amplification of HER2 in breast cancer serves as a notable example in this regard.

Thus, the primary goal of this study is to examine the feasibility of a NGS approach for detecting and quantifying SCNAs in cfDNA from CSF of patients with defined CNS cancers.

## Material and methods

### Study setting and patient cohort

**Fig. 1:**
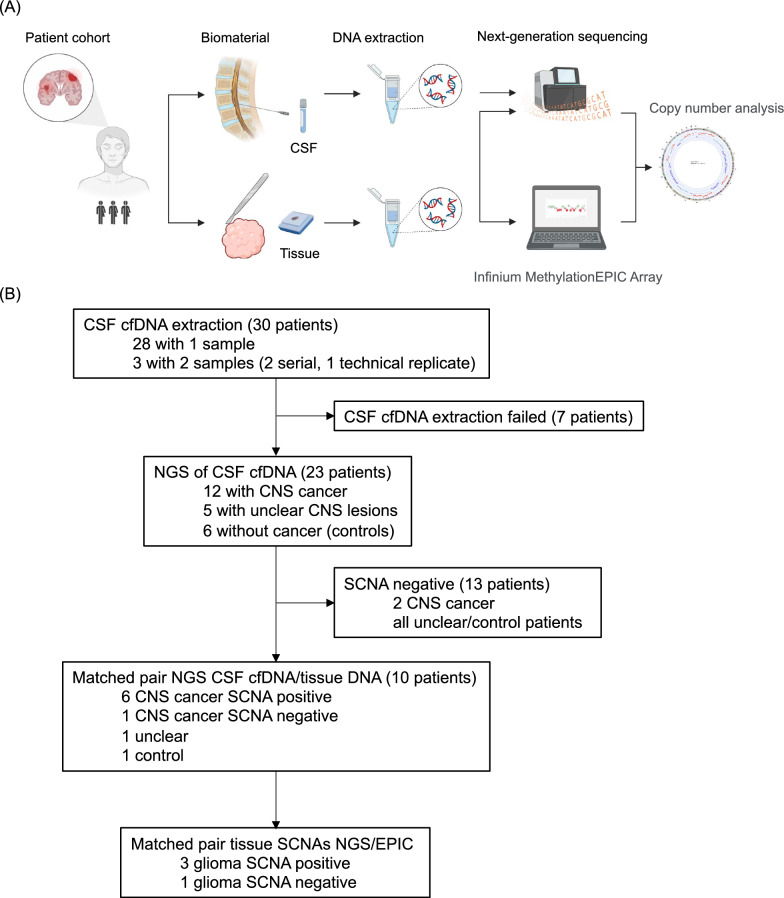
Study workflow and consort diagram. **A** Study workflow. CSF, cerebrospinal fluid; DNA, Deoxyribonucleic acid. **B** The consort flow diagram illustrates the study approach leading to a real-life cohort of patients with different types of CNS cancers with the purpose of analyzing somatic copy number aberrations (SCNAs) through next-generation sequencing

Within this systematic retrospective observational study, we collected 33 CSF samples from 30 patients **(**Fig. 1**, **Table 1). A sufficient yield of cfDNA for NGS was obtained in 26 samples collected from 23 patients. Of this NGS cohort, 12 patients had histologically confirmed CNS cancer, five patients had etiologically unclear CNS lesions, and six control patients lacked a cancer diagnosis. Among the 12 CNS tumor patients, nine patients suffered from primary CNS tumors such as gliomas (n = 6), CNS-DLBCL (n = 1) or meningeal melanocytoma (n = 1). Diagnoses of patients with secondary CNS tumors were brain and leptomeningeal metastases of lung adenocarcinoma (n = 1) and secondary CNS manifestation of lymphoma (n = 2). The median age at the time of CSF sampling was 54 years (range 22 to 85), with ten of 23 patients being female (43%). Serial CSF samples were obtained from two patients, with each patient undergoing two different lumbar punctures at different time points during the disease (samples 2 and 3 of patient 2; samples 21 and 22 of patient 19). All patients were diagnosed and treated at the University Hospital Frankfurt. Routine pathological or neuropathological workup of tumor tissue was performed at the Departments of Pathology and/or Neuropathology (Edinger Institute). Data from routine DNA methylation-based tumor profiling (Infinium MethylationEPIC Array) [10] was available from four patients in the Department of Neuropathology (Edinger Institute). Demographic and other clinical data (such as routine CSF parameters: protein, lactate, cytology) were extracted from patients’ records, deidentified, and entered into password-protected databases. All patients included in the study gave consent towards the sample collection and analyses. The study protocol was endorsed by the local ethical committee Frankfurt (SNO-9–2022).

**Table 1:** Patient characteristics of all patients included in next-generation sequencing

	Cancer (n = 12)	Non cancer (n = 11)
General		
Median age in years (range)	55 (32–81)	51 (22–85)
Gender women % (n)	33 (4)	55 (6)
Primary CNS tumors % (n)	75 (9)	NA
Glioma % (n)	50 (6)	
CNS-DLBCL % (n)	8 (1)	
Meningeal melanocytoma % (n)	8 (1)	
Secondary CNS tumors % (n)	25 (3)	NA
Secondary CNS lymphoma % (n)	17 (2)	
Brain Metastases % (n)	8 (1)	
LMD % (n)		
MRI-suspected % (n)	33 (4)	9 (1)
CSF cytology positive % (n)	42 (5)	0 (0)
CSF SCNA positive % (n)	83 (10)	0 (0)

### Collection and processing of cerebrospinal fluid

All CSF samples were collected during routine clinical care, primarily through lumbar puncture, except for two patients who underwent ventricular CSF sampling (patients 2 and 5). Samples were immediately centrifuged at 400 g for 10 min to separate cell-free from cellular components and transferred into liquid nitrogen for storage and shipped frozen to the central laboratory of Chronix (Göttingen, Germany).

### Extraction, quantification and quality control of cfDNA

The cfDNA was extracted from 33 CSF samples (1–2 mL) using the High Pure Viral Nucleic Acids Extraction Kit LV (Roche) as previously described for plasma specimen[19]. The entire processing, including cfDNA extraction and sequencing, was conducted on two separate aliquots from the CSF sample of patient 7. These aliquots (samples 8 and 9) were utilized as technical controls. The concentrations of the total extracted cfDNA were determined by droplet digital PCR as previously described for plasma specimen [19].

### Shallow whole-genome sequencing

Sequencing libraries were prepared for all samples that yielded ≥ 3 ng cfDNA using the SMARTer ThruPlex Kit (Takara) with 5–50 ng input depending on sample yield. Shallow whole-genome sequencing with ~ 39 M (SD: 15 M) read-pairs per sample was performed using a NextSeq500 (Illumina) instrument. Sequence data were mapped to the human reference genome (HG19) using BWA [22]. Duplicates and read-pairs with a mapping quality (MAPQ) < 60 were removed (average read-pairs after filtering: 20 M, SD: 8 M per sample). Additionally, DNA from matching Formalin-fixed Paraffin-embedded (FFPE) bulk tissue of 9 patients (7 with diagnosis of CNS cancer, 1 control patient, and 1 patient with an unclear CNS lesion) was sequenced accordingly (average read-pairs: 13 M, SD: 3 M), with 50 ng DNA used for sequencing library preparation.

### SCNA profiling

SCNAs were in 5.5 Mbp sliding bins (genomic windows). Depth-of-coverage analyses were conducted using the R package QDNAseq[23]. Briefly, after correction for GC-content and mappability per bin, the read counts were normalized by the median read count over all windows. This ratio was transformed into log2 ratios. Windows with log2-ratios above 0.1 for amplifications or below -0.1 for deletions were scored as significantly aberrant. Three metrics were developed to convert the detected SCNAs into a (semi-)quantitative diagnostic measure: first the absolute log2-ratios for bins above or below the significance limits were summed to give the overall CNI score; second, the number of significantly aberrant bins was counted and samples with ≥ 5 aberrant bins were deemed SCNA positive; and third, the CNI Score was divided by the number of significant bins resulting in the tumor-cfDNA fraction score. This measure served as a proxy for the level of tumor-derived cfDNA, as the aberrant log2-ratios in samples with higher tumor-cfDNA levels are less diluted by normal cfDNA resulting in a higher tumor-cfDNA fraction score. Furthermore, the tumor-cfDNA fraction score is independent of the amount and size of copy-number aberrations present in the individual tumor.

### Infinium MethylationEPIC Array of tumor tissue

SCNAs detected in tumor bulk DNA by NGS were compared to SCNAs acquired by EPIC array during neuropathological routine diagnostic. The FFPE tumor tissue was cut in 4µm thin sections mounted on glass slides and H&E stained. Punch biopsies from tumor cell densest areas were subjected to DNA isolation and bisulfite conversion. The DNA was hybridized onto the Human Methylation EPIC array according to the manufacturer’s protocol. The idat files from EPIC array of 4 patients were uploaded onto the molecularneuropathology.org website for tumor classification and visualization of methylome-based copy number alterations.

### Statistical analysis and data visualization

Detailed information on the statistical analyses is indicated in the figure legends or respective methods sections. Analyses were performed using JMP 16.2.0 and R (4.3.0). Illustrations were created with biorender.

### Use of large language models

ChatGPT was used exclusively for language editing of the article. After using this tool, the authors reviewed and edited the content as needed and take full responsibility for the content of the publication.

## Results

### Patient cohort and methodological feasibility

**Fig. 2:**
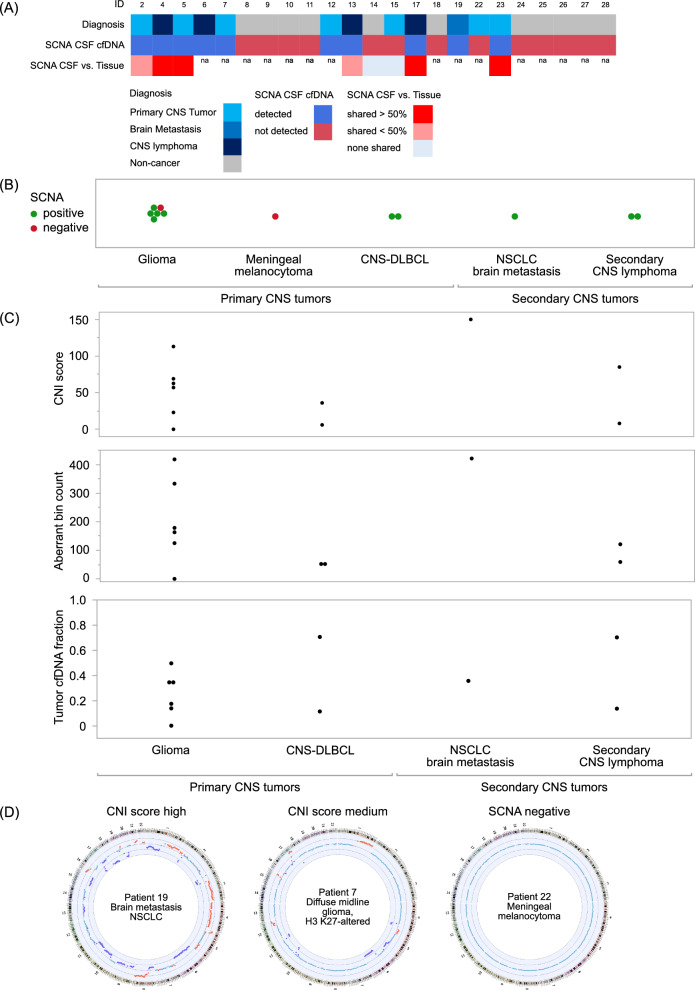
Somatic copy number aberrations in cell-free DNA from cerebrospinal fluid of patients with CNS cancers. **A** Overview clinical diagnosis and SCNA parameters. CSF cfDNA SCNA positivity vs. negativity and comparison of SCNA profiles in CSF and corresponding tissue (shared SCNAs) are depicted. **B** Frequency of detection of somatic copy number aberrations (SCNAs) across various CNS cancer types. SCNA positive samples are shown in green, negative samples in red. **C** SCNA parameters (CNI score, aberrant bin count, tumor cfDNA fraction) across the tumor patients. **D** Circos plots showing the copy number profile of cfDNA from cerebrospinal fluid of three exemplarily tumor patients with a high or medium CNI score in contrast to a patient without SCNAs

Extraction of cfDNA was conducted from 33 CSF samples obtained from 30 patients, including one patient with duplicate samples for technical control (revealing identical results) and two patients with serial CSF samples. While the CSF input volume varied from 0.8 to 3 ml, successful cfDNA extraction was achieved in 26 samples from 23 patients. Seven CSF samples (one control patient and six patients with histologically confirmed CNS tumors) did not yield sufficient cfDNA (> 3 ng) for NGS (Fig. 1**, **Table 1, Supplementary Table 1). Genomic coverage ranged from 0.19 × to 1.1x (Table 2). NGS-based cfDNA profiling enabled the detection of SCNAs, a hallmark of tumor-derived cfDNA, in CSF samples. This profiling also allowed for the comparison of SCNA profiles in cfDNA from CSF with those from matching tissue samples. Additionally, we evaluated the diagnostic relevance of each SCNA profile detected in CSF cfDNA and tissue, incorporating criteria for essential and desirable SCNAs based on the current WHO classification of CNS tumors for the patients with primary brain tumor diagnoses (overview in Fig. 2A, Supplementary Table 1).

**Table 2:** Sequencing metrics of CSF samples

	Mean (min–max)
CSF cfDNA quantity mean in ng	182 (3 to 1394)
Mapped reads (% of total reads)	0.95 (0.69–0.98)
Coverage depth mean	0.5x (0.19–1.1)

### Sensitivity and specificity of ctDNA detection in CSF

Ten out of 12 CSF samples from CNS cancer patients scored SCNA-positive (83%) and all control samples from patients with benign diagnoses (n = 6) or unclear CNS lesions (n = 5) were SCNA-negative (Table 1, Supplementary Fig. 1, Supplementary Table 1). SCNAs in CSF cfDNA were observed in seven out of nine patients with primary CNS tumors and in all three patients with secondary CNS tumors (Fig. 2, Table 3, Supplementary Fig. 1). SCNAs were detected in six out of 12 (50%) patients at the time of first diagnosis and in 6 out of 12 (50%) patients during progressive diseases (PD) stages (Supplementary Fig. 2). Among the ten SCNA-positive tumor patient samples we observed a mean CNI score of 58 (range 6 to 150), a mean count of aberrant bins of 185 (range 51 to 421), and a mean tumor fraction score of the cfDNA of 0.34 (range 0.11–0.71) (Fig. 2, Table 4, Supplementary Table 1).

**Table 3:** Patient characteristics of tumor patients with SCNA positive and SCNA negative cfDNA profiles in next-generation sequencing

	CSF SCNA positive cancer patients (n = 10)	CSF SCNA negative cancer patients (n = 2)
Primary CNS tumors % (n)	70 (7)	100 (2)
Glioma % (n)	50 (5)	50 (1)
CNS-DLBCL % (n)	20 (2)	0 (0)
Meningeal melanocytoma % (n)	0 (0)	50 (1)
Secondary CNS tumors % (n)	30 (3)	0 (0)
Secondary CNS lymphoma % (n)	20 (2)	0 (0)
Brain Metastases % (n)	10 (1)	0 (0)
LMD % (n)		
MRI-suspected % (n)	30 (3)	50 (1)
CSF cytology positive % (n)	50 (5)	0 (0)

**Table 4:** SCNA characteristics of all tumor patients with SCNA positive cfDNA in next-generation sequencing

	CNI score mean (range)	Aberrant bin count mean (range)	Tumor cfDNA fraction score mean (range)
SCNA positive patients (n = 10)	61 (6–150)	191 (51–421)	0.35 (0.11–0.71)
Primary CNS tumors (n = 7)	52 (6–113)	187 (51–418)	0.33 (0.11–0.71)
Glioma (n = 5)	65 (23–113)	241 (98–418)	0.30 (0.14–0.50)
CNS-DLBCL (n = 2)	NA (6–36)	NA (51–53)	NA (0.11–0.71)
Secondary CNS tumors (n = 3)	81 (8–150)	200 (59–421)	0.40 (0.14–0.70)
Secondary CNS lymphoma (n = 2)	NA (8–85)	NA (59–121)	NA (0.14–0.70)
Brain Metastases (n = 1)	NA (150)	NA (421)	NA (0.36)

**Fig. 3:**
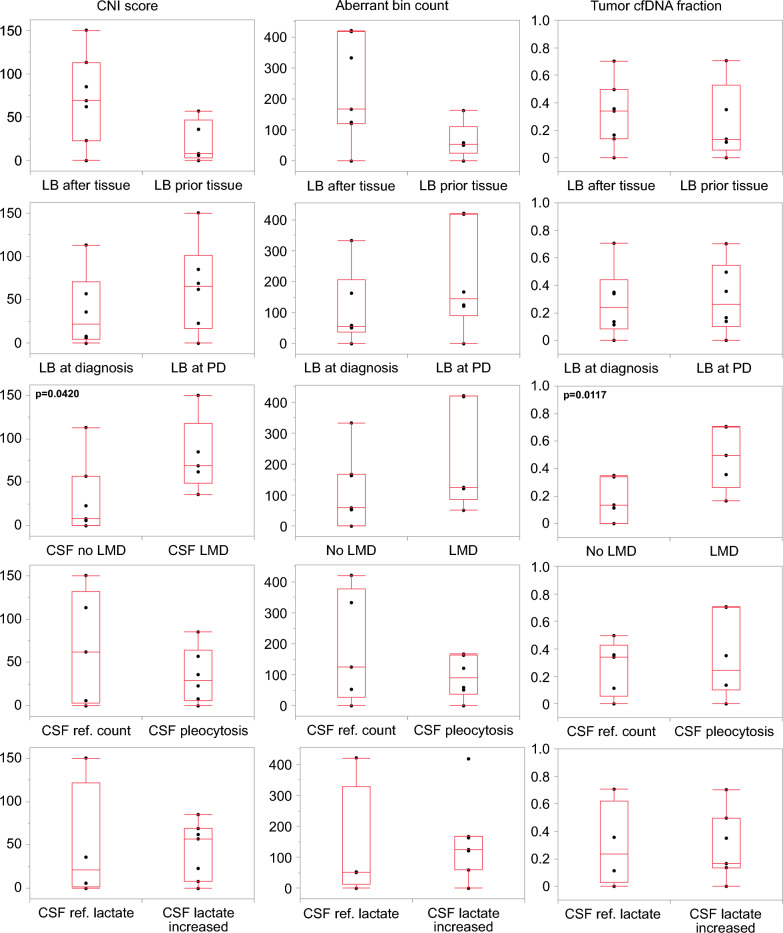
Correlation of CSF liquid biopsies with sampling and clinical variables. Box-plots depict the distribution of the NGS parameters (CNI score, aberrant bin count, tumor cfDNA fraction) considering defined sampling and clinical variables. Significant *p*-values < 0.05 are shown in the charts, otherwise no statistically significant differences were observed. LB = liquid biopsy; PD = progressive disease, LMD = leptomeningeal disease, cytology-confirmed; ref. = reference; CSF = cerebrospinal fluid.

Next, our aim was to investigate the potential impact of sampling and clinical variables on SCNA status, CNI score, aberrant bin count, and estimated tumor cfDNA fraction. Specifically, we compared the timing of CSF sample collection in relation to tissue sample collection, as well as the CSF collection timepoint in comparison to the disease stage (at the time of diagnosis, during PD, or during surveillance). The total count of abnormal genomic regions (aberrant bin count) and the CNI scores tended to be higher in CSF samples collected after tissue preservation, despite similar fractions of tumor cfDNA. However, there was also significant variation observed across the samples **(**Fig. 3**)**.

Regarding the rates of detecting SCNAs per se in CSF, seven out of 12 (58%) were observed after tissue collection for tumor diagnosis, and five out of 12 (42%) were detected prior to tissue collection (Supplementary Fig. 2). Concerning the potential impact of disease progression, there was a tendency towards higher CNI scores and abnormal genomic region counts in patients with progressive tumors compared to those with initial diagnoses. However, these differences did not reach statistical significance in our relatively small cohort **(**Fig. 3**)**.

### SCNA profiling of CSF cfDNA augments the opportunities of CSF cytopathology

Moreover, we examined the correlation of NGS parameters with cytology confirmed LMD, defined by the detection of tumor cells in the CSF sample during routine cytopathological assessment. While SCNAs were detectable in CSF cfDNA from all five patients with cytology-confirmed LMD, we additionally identified SCNAs in the CSF cfDNA from five out of seven patients without cytology-confirmed LMD (Fig. 3, Supplementary Fig. 2). The five patients with confirmed LMD demonstrated significantly higher CNI scores and fractions of tumor cfDNA compared to patients without LMD in cytology. Changes observed in routine CSF parameters, such as cell count and lactate levels, did not exhibit a significant correlation with the NGS parameters **(**Fig. 3**)**.

### Utility of SCNA profiling of cfDNA from CSF for diagnostic classification and disease monitoring of patients with CNS tumors

**Fig. 4:**
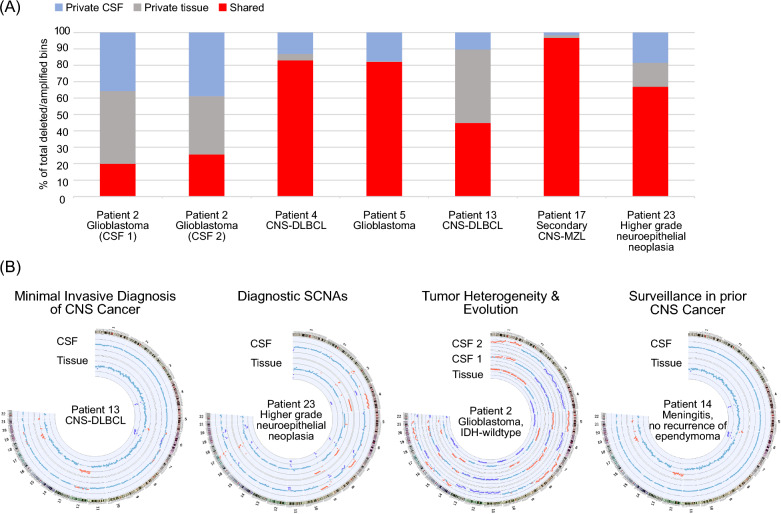
Diagnostic value of SCNA profiling of cell-free DNA from cerebrospinal fluid of patients with CNS cancers. **A** Concordance analyses between CSF and tumor tissue. Depicted is the fraction of SCNAs private to CSF (blue), private to tissue (gray) or shared between the two (red). **B** Circos plots with copy number profiles of CSF cfDNA compared to tissue DNA exemplifying the usefulness of SNCA profiling for minimal invasive detection of CNS cancer, molecularly informed diagnostic assessment, mapping of tumor heterogeneity and tracking tumor evolution as well as surveilling patients with a previous cancer diagnosis

Ultimately, we assessed the effectiveness of our CSF LB approach using a recently introduced set of quality criteria tailored to assess the benefits of LB tools in patients with brain tumors. Our evaluation focused on key aspects, including: (i) establishing a diagnosis and/or identifying diagnostically relevant genomic alterations (including copy number alterations incorporated as essential or desirable criteria in the current WHO classification [11]), (ii) monitoring tumor response to therapy, and (iii) tracking tumor evolution [8]. To address these inquiries, we complemented NGS of matching tissue samples, allowing for a direct comparison of SCNA profiling of CSF cfDNA and tissue DNA. Tissue was available for seven tumor patients (patients 2, 4, 5, 13, 15, 17, and 23), one patient with meningitis and a history of ependymoma (patient 14), and one patient with IgG4-associated orbital inflammation (Figs. 1, 2A and 4, Supplementary Fig. 3).

First, we aimed to determine whether tissue SCNAs could be traced in cfDNA from CSF, given the essential value of SCNA profiling in neuro-oncology [11]. A concordance analysis, comparing SCNA profiles directly between CSF and tissue, was conducted on six tumor patients with matched tissue/CSF pairs exhibiting SCNAs in their CSF cfDNA (patients 2 (two CSF samples), 4, 5, 13, 17, and 23). Shared SCNAs between CSF cfDNA and tumor tissue DNA were observed in all patients, as particularly notable in patients 4, 5, 17 and 23 (Fig. 2A, Fig. 4A, Supplementary Fig. 3).

To refine the diagnostic utility of our approach, we assessed defined diagnostic genomic alterations, and thereby also considered the criteria of the current WHO classification of CNS tumors for essential and desirable SCNAs in particular brain tumor subtypes, as outlined with positive results in several patients (Supplementary Table 2).

Besides providing relevant molecular pathological information, our data suggests that tracing SCNAs in CSF samples offers an avenue for expediting and facilitating the diagnostic process in patients through a less invasive approach as especially evident in patients with CNS lymphomas (patients 4, 6, 13, 17), a CNS tumor for which nonsurgical treatment is inherently preferred (Fig. 4B, Supplementary Fig. 1).

Beyond that, SCNA profiling of CSF holds the potential for mapping tumor heterogeneity and tracking tumor evolution (Fig. 4B, Supplementary Fig. 3). In fact, the concordance analysis also unveiled distinct SCNAs exclusive to either CSF or tissue in all patients, albeit generally to a lesser extent than shared SCNAs between CSF and tissue (Fig. 4A, Supplementary Fig. 3). SCNA variations between CSF and tissue can be attributed either to spatial heterogeneity within a tissue sample or tumor evolution throughout the disease. Evolution of the SCNA profile could be traced in patient 2 with glioblastoma where copy number profiling of the tissue biopsy at first diagnosis in comparison to the CSF LBs at PD overspun a disease course of approximately 1.5 years including multimodal glioblastoma treatment. The two serial CSF LBs at PD collected within only one week did not reveal major differences compared with each other (Fig. 4). As an example for tracing stable disease (SD) over a longer period, serial CSF LBs of patient 19, with brain and leptomeningeal metastases from lung adenocarcinoma, showed a stable CSF profile over a 10-month interval (Supplementary Fig. 1B). To assess the tool’s applicability in monitoring disease activity or recurrence after complete remission, we sequenced CSF cfDNA from patients under surveillance due to a previous history of CNS cancer and uncertainty regarding the differentiation between tumor recurrence and other potential causes of clinical deterioration (patients 12 and 14): The CSF LB supported the recurrence of pleomorphic xanthoastrocytoma in patient 12 (Supplementary Fig. 3), whereas favoring the diagnosis of a postoperative infection over tumor recurrence in patient 14 after curative resection of posterior fossa ependymoma (Fig. 4, Supplementary Fig. 3).

Of note, SCNA differences between CSF and tissue can also be attributable to methodological aspects: Patient 15, diagnosed with glioblastoma and displaying typical copy number alterations, had no detectable SCNAs in CSF cfDNA collected 1.5 weeks prior to surgical tissue resection. This suggests a deficiency of tumor-derived cfDNA at relevant levels in this CSF sample (Supplementary Fig. 3). This scenario highlights that a SCNA-negative CSF sample does not fully rules out the diagnosis of CNS cancer. Notably, in terms of the diagnostic value of SCNA-negativity, no SCNAs were detected in non-cancer CSF samples, as also evidenced by the matching tissue and CSF samples from a patient with confirmed histology of IgG4-associated orbital inflammation (Supplementary Fig. 3). From a technical perspective, it is noteworthy that the SCNA data obtained through methylation-based profiling of tumor tissue from four glioma patients showed no significant disparities compared to SCNA profiling via NGS (Supplementary Fig. 4). Additionally, a technical replicate of independently processed CSF aliquots from patient 7 exhibited no SCNA deviations between the aliquots, as expected (Supplementary Table 1).

## Discussion

Precise diagnostic classification and effective monitoring of therapy response and resistance is crucial for improving the prognosis of patients with CNS cancer. CSF LBs demonstrate high sensitivity in detecting genomic alterations of tumor cfDNA [18]. CSF LBs are particularly beneficial when the affected CNS areas are functionally important, the patient’s overall condition is compromised, or the brain tumor type is not amenable to surgical intervention. Such scenarios include CNS lymphomas per se, but also gliomas or brain metastases located in challenging brain regions, or when distinguishing non-neoplastic from potentially neoplastic CNS lesions. Further, CSF LBs allow for repeated sampling to guide patient-centered clinical decision making. Here, we describe shallow NGS of CSF cfDNA as tool for the detection of diagnostically relevant SCNAs in brain tumor patients (Fig. 1**)**.

The detection of SCNAs in cfDNA is highly specific for tumor-derived cfDNA, with no false positive SCNAs observed in the control patients without a cancer diagnosis in our cohort, aligning with findings from other studies in this field. As a hallmark of tumor-derived cfDNA, SCNAs were identified in the CSF cfDNA of the majority (83%) of patients with CNS tumors (Fig. 2). Of note, SCNAs were also in cfDNA of tumor patients without cytopathological tumor cell detection in CSF (Fig. 3, Supplementary Fig. 2) supporting that CSF cfDNA, regardless of cytopathology-confirmed tumor cells, can contain tumor-derived cfDNA [6]. In terms of further assessing key aspects of effectiveness of our LB tool [8], we were able to trace a substantial number of shared SCNAs between tissue and CSF (Figs. 2A, 4) as well as diagnostic SCNAs [11] (Supplementary Table 2). Additionally, we identified prognostically relevant SCNAs like on the long arm of chromosome 6 in CNS lymphoma patients (Supplementary Fig. 1) [24] associated with an aggressive clinical course [21]. Differences in SCNAs between tissue and CSF can also be attributed to tumor evolution as traced in the longitudinal biomaterial analyses throughout the disease course of patient 2 with glioblastoma (Fig. 4). Further, SCNA profiling of CSF cfDNA proved advantageous for surveillance of patients with prior history of CNS cancer and the need to distinguish tumor recurrence from other causes of clinical deterioration (e.g. patients 12, 14) (Supplementary Fig. 1). Taken together, our approach proved valuable in supporting or establishing diagnoses faster and less invasively in CNS tumor patients (Fig. 2A, Supplementary Table 1, Supplementary Fig. 3).

In addition to assessing the mere SCNA profile, our approach offers the possibility to quantify chromosomal instability holding potential for correlation with outcome parameters [19]. While we observed a wide range of SCNA dimensions in CSF cfDNA, as indicated by the CNI scores and the total count of abnormal genomic regions (aberrant bin count), there was a tendency towards higher CNI scores and aberrant bin counts in patients whose CSF was collected after tissue preservation compared to those collected prior to tissue preservation (Fig. 2). Here, it is important to note the possibility of tumor cfDNA carrying over into the CSF during surgical tissue collection. However, the mere detection rate of SCNAs was independent of the timing of tissue collection. Further, patients with PD stages showed a trend towards higher CNI scores and aberrant bin counts compared to patients with initial diagnoses suggesting a potential association of these parameters with the disease stage (Fig. 3). Of note, most of these analyses did not reach statistical significance and must be interpreted with caution. The limited cohort size prevents us from determining parameters such as frequency rates of SCNA positivity or a systematic comparison of SCNA patterns across different types of CNS cancer.

Of note, SCNA profiling relies on (i) the release of tumor cfDNA into the CSF and (ii) the presence of SCNAs in tumor-derived cfDNA. Consequently, the applicability of this LB approach has limitations in case of few or copy number-neutral genomic alterations. In case of low levels of tumor-derived cfDNA within the total quantity of cfDNA in a CSF sample, the detectability of SCNAs can reach its limits. Thus, although the detection of SCNAs is highly specific for tumor-derived cfDNA, the absence of SCNAs in CSF cfDNA does not conclusively indicate that the tumor is SCNA-negative. Subtle alterations can fall below the level of detection and the LB may not capture tumor-derived SCNAs or their full spectrum as in patients 13 or 15 (Supplementary Fig. 3). To overcome this limitation, novel LB approaches aim to enhance the sensitivity of detecting cancer-related genomic alterations by combining low pass NGS pan-cancer assays (for SCNA detection) with targeted panel-based sequencing to additionally capture disease-specific gene fusions and mutations of cfDNA [25].

In summary, our findings support the utility of SCNA profiling of cfDNA from CSF in defined CNS cancers, despite the constraints of a retrospective exploratory study with a relatively small sample size. SCNA profiling of CSF cfDNA could have expedited the diagnostic process and the initiation of tumor-specific therapies. This was particularly evident in cases lacking cytology confirmed LMD or those with inconclusive findings from stereotactic biopsies. Furthermore, it demonstrated its potential for minimally invasive mapping of tumor heterogeneity and tracking tumor evolution. However, like other promising pilot studies on emerging LB techniques [5–8, 26–28], our study faces the common limitation of lacking standardization, validation, and methodological harmonization necessary for the successful integration into routine clinical practice. Key considerations include the technical and methodological applicability, resource-consciousness and economic feasibility. Thus, to fully comprehend the potential prognostic and predictive value of SCNA profiling of CSF cfDNA, validation through larger prospective studies is warranted [5–8]. Standardized sequential CSF profiling at defined time points before, during, and after therapeutic interventions, would enhance our understanding of the utility of cfDNA SCNA profiling for disease monitoring. Notably, the potential of CNI scoring of plasma LBs to predict responses to immunotherapy in non-CNS tumors [19] should be evaluated for brain tumor patients by CSF profiling. This is particularly relevant given the increasing role of immunotherapeutic approaches in secondary [29], but also primary CNS tumors (as evidenced by several trials such as CheckMate 498 [30] and 548 [31], or advanced immunotherapies like the NOA-16 [32] or the CAR2BRAIN [33] trial) which underscore the need for reliable biomarkers for monitoring (immune) therapy responses and offering insights into resistance mechanisms.

## Conclusions

Taken together, our study supports exploration of SCNA profiling of cfDNA from CSF of brain cancer patients. Further large-scale prospective trials, incorporating serial sampling, are necessary to fully decipher its translational value. Despite the challenges discussed, we maintain an optimistic outlook that emerging technologies [15, 16, 25] will contribute to the improvement and applicability of novel diagnostic LB tools soon.

## Supplementary Information

Additional file 1.

Additional file 2.

Additional file 3.

## Data Availability

Data generated or analyzed during this study are included in this article and its supplementary information files. Additional relevant data is available on reasonable request.

## Abbreviations

CNS: Central nervous system
CSF: Cerebrospinal fluid
cfDNA: Cell-free DNA
CNI: Chromosomal number instability
CNS-DLBCL: Diffuse large B-cell lymphoma of the CNS
LMD: Leptomeningeal disease
LB: Liquid biopsy
MRI: Magnetic resonance imaging
NGS: Next-generation sequencing
PD: Progressive disease
SCNAs: Somatic copy number aberrations
SD: Stable disease
