# Supplementary material for: Detection of diagnostic somatic copy number alterations from cerebrospinal fluid cell-free DNA in brain tumor patients
Source: Acta Neuropathol Commun. 2024 Nov 20;12:177. doi: 10.1186/s40478-024-01887-9 (PMC11580493; doi:10.1186/s40478-024-01887-9)
Supplement: Supplementary file 1 — Additional file 1. [file 40478_2024_1887_MOESM1_ESM.docx]

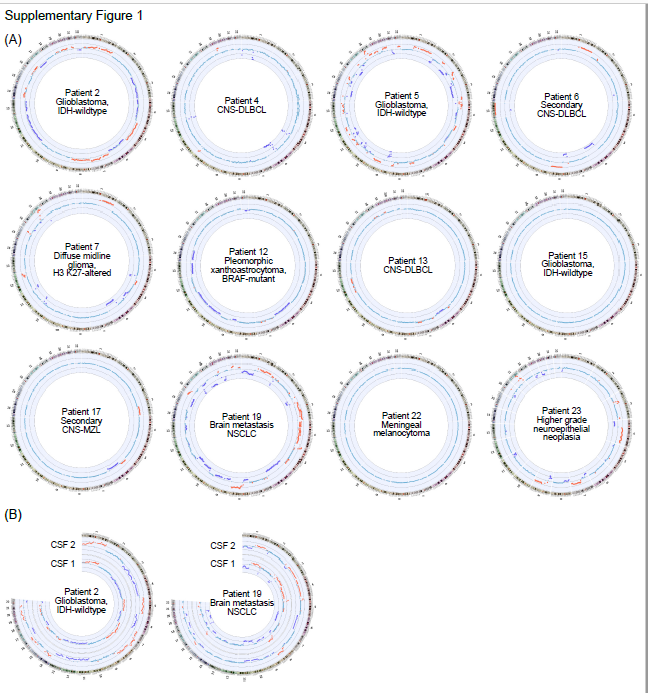


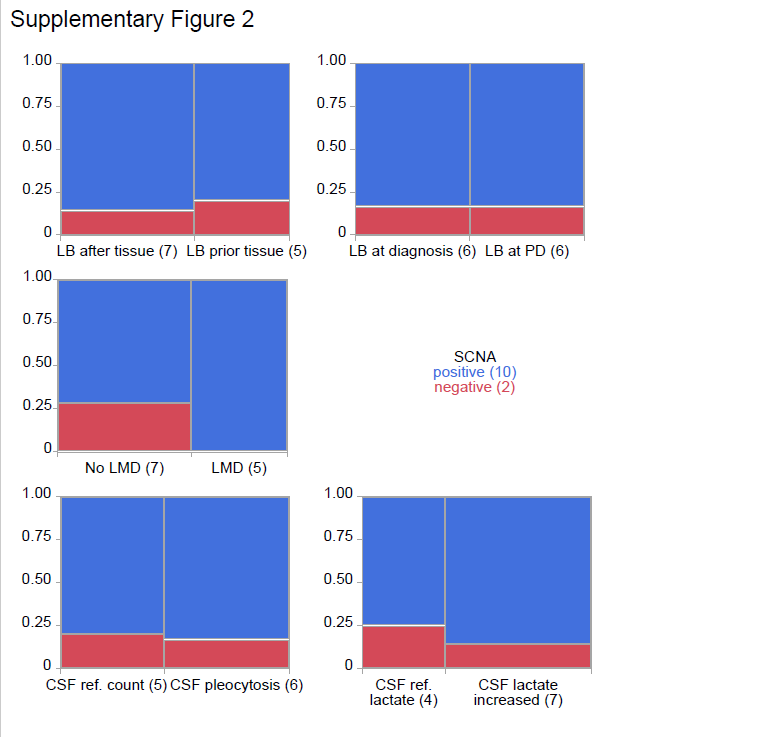


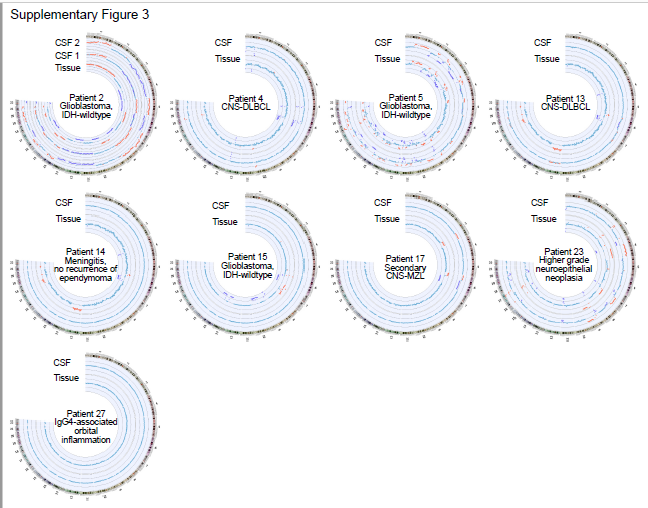


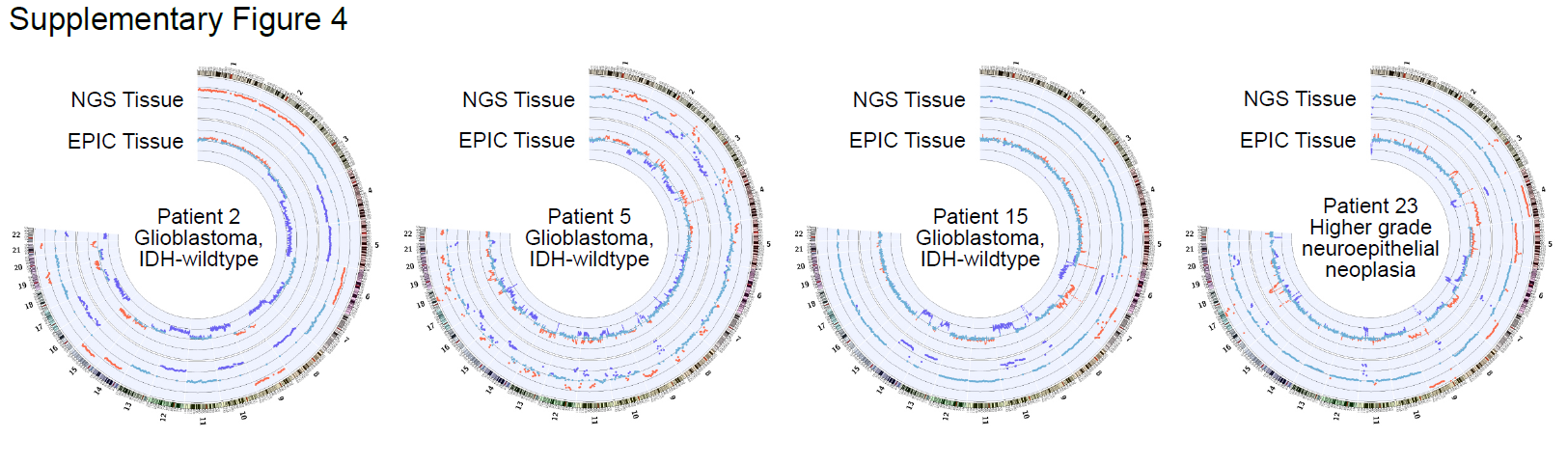


**Supplementary material**

**Supplementary Figure 1:** Copy number profiles of tumor patients with somatic copy number aberrations in cell-free DNA from cerebrospinal fluid.

(A) Circos plots showing the CNI values of significantly aberrant bins in cfDNA from cerebrospinal fluid of all twelve tumor patients. (B) Combined circos plots of two CSF samples obtained at different time points from patients 2 and 19.

**Supplementary Figure 2:** Correlation of SCNA detection with sampling and clinical variables.

Proportions of tumor patients with (blue) vs. without (red) detection of somatic copy number aberrations (SCNAs). LB = liquid biopsy; PD = progressive disease, LMD = leptomeningeal disease, cytology-confirmed; ref. = reference; CSF = cerebrospinal fluid.

**Supplementary Figure 3:** Copy number profiles in cell-free DNA from cerebrospinal fluid as compared to DNA from tissue.

Combined circos plots showing the copy number profile of cfDNA from cerebrospinal fluid (CSF) in contrast to DNA from tissue samples of all patients available for this matched pair approach.

**Supplementary Figure 4:** Copy number profiles of DNA from glioma tissue obtained by either NGS or EPIC array.

Combined circos plots showing the copy number profile of DNA from glioma tissue obtained either by NGS or by EPIC array of all patients where an EPIC array was performed during clinical workup.

**Supplementary Table 1:** Detailed patient characteristics of the total cohort.

**Supplementary Table 2:** Diagnostic relevance of genomic alterations.
